# Does low BMI affect ART outcomes?

**DOI:** 10.5935/1518-0557.20180021

**Published:** 2018

**Authors:** João Batista A Oliveira

**Affiliations:** 1 Center for Human Reproduction Prof Franco Jr., Ribeirão Preto, SP, Brazil

Several studies have reported that elevated body mass index is associated with a need for higher drug doses to stimulate ovulation, changes in oocyte morphology, reduction in fertilization and implantation rates, reduced rates of clinical pregnancy and live births, and increased miscarriage rates. These findings have led to growing concerns regarding obese or overweight women undergoing infertility treatment. However, the other extreme of body weight, underweight (body mass index/BMI <18.5), also warrants attention.

Unfavorable pregnancy outcomes and infertility problems have been reported in women with low body weight. However, evidence of the effects of low BMI on ART outcomes is conflicting. On one hand, low BMI has been associated with a reduced probability of achieving pregnancy, increased risk of miscarriage and reduced live birth rate (Wang *et al.*, 2000; Wittemer *et al.*, 2000; Veleva *et al.*, 2008; Singh *et al.*, 2012; Cai *et al.*, 2017); on the other hand, several studies have found no effect on treatment outcomes (Lashen *et al.*, 1999; Fedorcsák *et al.*, 2004; Li *et al.*, 2010; Provost *et al.*, 2016; MacKenna *et al.*, 2017). Nevertheless, some studies report that patients with low BMI have worse clinical outcomes than patients with normal BMI, although these results were not statistically significant.

The relatively small sample size of underweight groups may explain the variation among studies and hinder statistical evaluation. Another factor that could explain the heterogeneity of outcomes is the influence of biological differences such as ethnicity. Indeed, an interaction between race and BMI has been described, suggesting that this may be a significant limitation in the interpretation of results (Luke *et al.*, 2011). Provost *et al.* (2016) found that increasing BMI seemed to have a detrimental effect on IVF outcomes, but the opposite was observed in underweight patients. However, the authors noted that one of the limitations of the study was the inability to adjust for patient race across BMI categories. In a study conducted in a Latin American population, MacKenna *et al.* (2017) did not observe a correlation of low weight with IVF outcomes. However, Cai *et al.* (2017) reported that low BMI was associated with reduced live birth rates and increased miscarriage rates compared with normal weight in a Chinese population after controlling for important covariates known to influence IVF outcomes.

Although more studies are needed to elucidate the real effect of low BMI, weight counseling before starting ART cycles may be useful for underweight patients. However, the specific characteristics of the populations being treated and results from each clinic should be taken into account.
